# Efficient merging and validation of deep learning-based nuclei segmentations in H&E slides from multiple models

**DOI:** 10.1016/j.jpi.2025.100443

**Published:** 2025-04-15

**Authors:** Jagadheshwar Balan, Shannon K. McDonnell, Zachary Fogarty, Nicholas B. Larson

**Affiliations:** Quantitative Health Sciences, Mayo Clinic, Rochester, MN, USA

**Keywords:** Digital pathology, Nuclei segmentation, Gene expression, Cell type proportions, Deep-learning, Multiomics

## Abstract

Characterizing cellular composition in tissue samples offers fundamental insights into functional and biological processes. Understanding the abundance or lack of specific cell types, such as inflammatory cells in the context of microenvironments such as tumor can help guide disease progression and personalized medicine. Several clinical laboratory methods to characterize the cellular composition are limited by scalability and high-costs. Digitizing pathology slides and applying deep learning (DL) models have enabled efficient and cost-effective nuclei segmentation and cell type quantification; however, the DL-models are limited by their inability to segment specific cell types and specific models may be more effective than others at certain tasks. Consequently, there remains a need for methods that leverage the strengths of multiple models to efficiently integrate nuclei segmentation for various cell types. In this study, we propose a novel solution for integrating nuclei segmentation from multiple DL-methods on hematoxylin and eosin slides from 471 normal prostate samples and highlight the limitations of using a single DL-method. We validate the DL-derived cell type proportions, by comparing against estimates from a manual pathologist review and show that the integrated approach results in higher concordance over the individual models. We further validate the derived cell type proportions from the DL-methods by their ability to explain the variance of RNA gene expression. The integrated approach yields robust cell type proportions that explain the variance of the gene expression with 12% and 22% relative improvement than current state-of-the-art model and manual pathologist review, respectively. The subset of 403 genes with high explained variation (>30%) by epithelial proportion were significantly enriched for relevant biological pathways. These findings indicate that ensemble approaches to nuclei segmentation and cell-type classification may provide more accurate representations of cellular composition from digitized slides.

## Highlights

• Deep learning (DL) methods allow understanding of cellular composition in H&E slides.
• DL models have strengths and weaknesses that vary by cell types, owing to their training sets.
• Study presents an approach to efficiently merge nuclei segmentation on H&E slides from DL models.
• Resulting cell type proportions show improved concordance to manual pathologist review.
• Validating the cell proportions with gene expression underpins utility of merged segmentations.

## Graphical abstract

**Figure:**
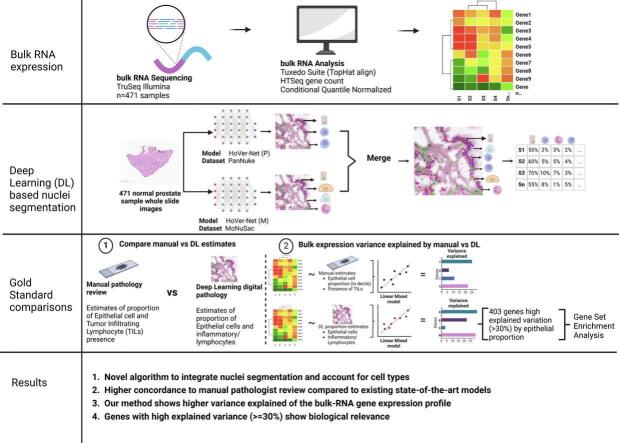

## Introduction

Laboratory methods for studying cell-type composition and gene expression by cell types involve usage of immunohistochemistry and flow cytometry; however, these methods lack the ability to probe multiple genes and cell types in parallel.^23^ With the rapidly evolving field of single cell RNA sequencing (scRNA-seq), quantification of multiple cell types and corresponding characterization of cell-specific gene expression have remarkably advanced.^33^ However, scRNA-seq remains expensive, requires high-quality tissue samples, and presents numerous analytical challenges.^16^, ^33^ With large bulk RNA-seq datasets readily available for various tissues and conditions, new algorithmic approaches have emerged for expression deconvolution of bulk RNA-seq using well-defined scRNA-seq reference panels.^4^, ^7^, ^8^, ^15^, ^16^ However, these panels may not be available on relevant cell types nor fully capture expression profile variability. With the broad availability of bulk RNA-seq and complementary data types, novel and innovative approaches are needed to fully use these existing data resources. Widespread availability of corresponding pathological hematoxylin and eosin (H&E)-stained slides for tissue-based bulk RNA-seq and the ease of slide digitization enables an alternative approach to characterize and quantify cell types using deep learning (DL). Digitized slides available as a whole slide image (WSI) are comprised of thousands of pixels that represent various nuclei corresponding to several cell types and the extracellular matrix. Identification of cell types on WSIs using DL methods has advanced in recent years by applying specialized neural network architectures involving convolutional neural networks (CNNs) and generative adversarial networks (GANs).^10^, ^12^, ^18^, ^26^, ^36^ StarDist is a popular nuclei segmentation method which successfully performs nuclei segmentations; however, it requires further custom model training for identifying the various cell types.^26^ Similarly, a conditional GAN-based Multi-Organ Nuclei Segmentation method identifies cells and segments nuclei but requires further custom model training and tuning to accurately identify the various cell types.^21^ Custom model training, tuning, and deployment are often very resource heavy, and time consuming. Additionally, DL methods are highly reliant on the robustness of the underlying training datasets and they lack applicability in accounting for diverse cell types.^12^ Hence, there is a need for efficient methods that account for segmenting and classifying various cell types.

In this study, we explore novel and efficient methods to estimate cell type proportions by integrating nuclei segmentation results from multiple DL methods and examine the reliability of the estimated proportions using digitized pathological H&E slides from a large collection of normal prostate tissue samples. We additionally validate the robustness of the cell type predictions using gold-standard datasets that include manual pathologist review and bulk-RNA gene expression profiles.

## Methods

### Sample cohort selection

**Fig. 1:**
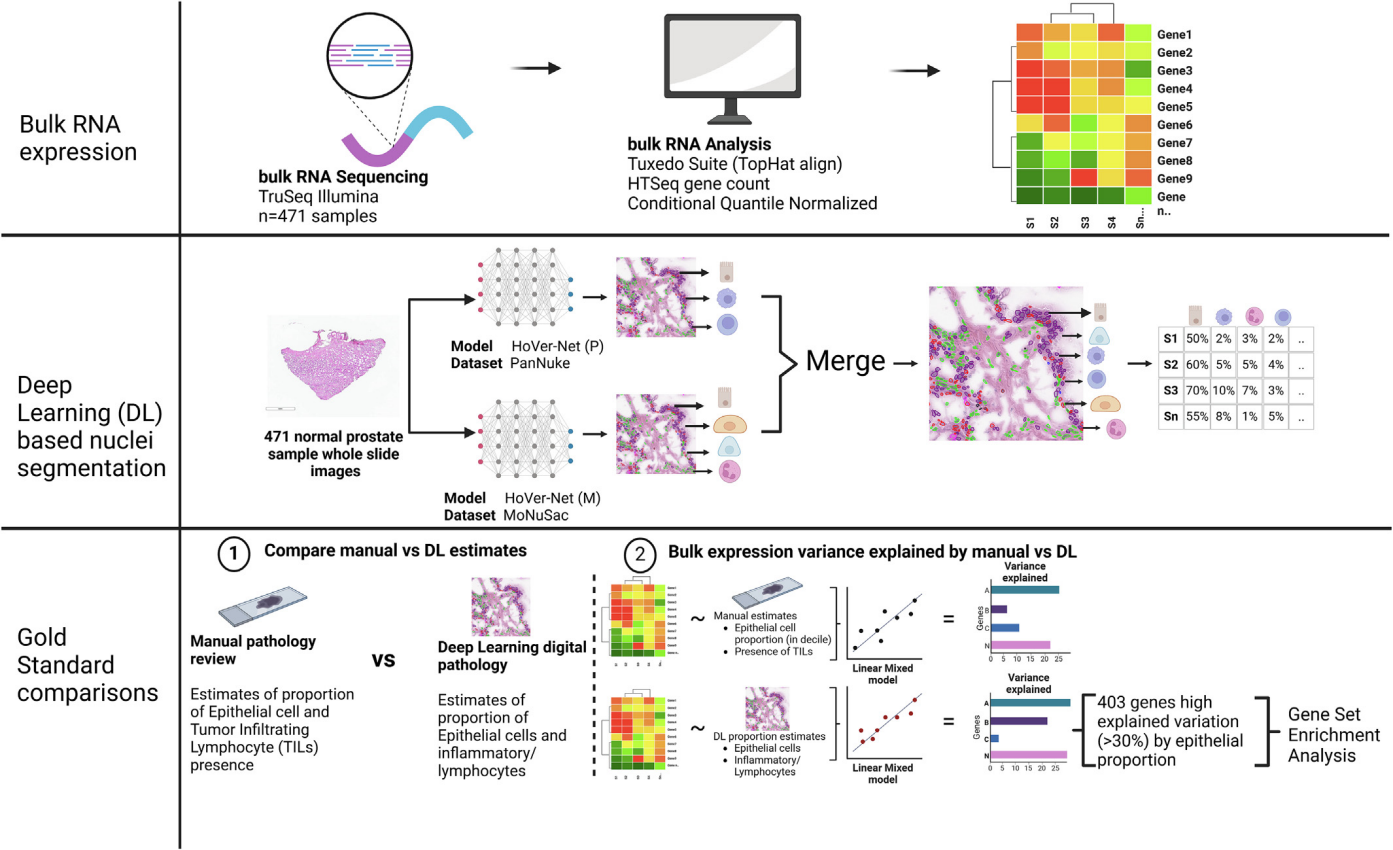
Bulk RNA expression profiles are generated using an in-house pipeline and then the cell proportion profiles are generated using deep learning (DL) methods trained on multiple datasets and the segmented predictions are merged. Gold-standard comparisons are performed between estimates from manual pathologist review and DL methods. Additionally, a linear mixed model is built to predict bulk RNA expression data using proportions from manual and DL methods to understand the variance of expression data explained. Subsequently, gene set enrichment analysis (GSEA) is performed with 403 genes with high explained variance from the DL methods to evaluate biological relevance. (Created in BioRender. Balan, J. (2024) https://BioRender.com/e11g294). (For interpretation of the references to color in this figure legend, the reader is referred to the web version of this article.)

An overview of our study, the dataset, and methods are detailed in Fig. 1. Our analyses leverage data generated from a previous study aiming to identify gene expression quantitative trait loci in normal prostate tissue.^27^ Briefly, fresh frozen prostate tissue samples were acquired from an archive collection of material from radical prostatectomy and cystoprostatectomy patients. New H&E slides were prepared from each tissue sample for additional expert pathologist review. Inclusion criteria were defined based on these reviews to carefully select normal prostate tissue and remove low quality samples. The inclusion criteria required absence of prostate cancer, absence of high-grade prostatic intraepithelial neoplasia and benign prostatic hyperplasia, normal prostatic epithelial glands representing ≥40% of all cells and lymphocytic population representing ≤2% of all cells.^27^

### Manual pathology review of H&E slides

The manual pathology review on all the H&E slides of the 471 normal prostate samples was performed as part of a previously published study.^27^ Briefly, the pathologist provided crude estimates of epithelial cells on a per sample basis. Manual pathological review is limited by the ability to scale, owing to the presence of several thousand nuclei on the whole slide. Hence, the manual pathologist review yielded crude estimates of epithelial cells in deciles (e.g., 40%, 50%). The manual pathology review also involved a careful inspection to confirm that the slides had a lymphocytic population representing ≤2% of all cells. Finally, the pathologist review also yielded information on cases that have presence of higher tumor infiltrating lymphocytes (TILs).

### RNA sequencing and bioinformatics

RNA extraction, quality assessment, library preparation, sequencing, base calling, and post-sequencing quality control were performed as previously described.^27^ Briefly, the analyses included using RSeqQC for analyzing quality of samples, MAPRSeq for the secondary analysis, and HTSeq for gene counts.^3^, ^17^, ^20^, ^28^, ^35^ Gene counts were normalized using the conditional quantile normalization method. These data are publicly available via dbGaP (accession phs000985.v2.p1).

### Whole slide digitization

Multiple H&E slides were available per sample by initial study design, and a representative H&E-stained slide adjacent to the tissue slices used for bulk RNA sequencing experiments was digitized using the Aperio Leica GT-450 digital pathology scanner platform with a resolution of 40× magnification and 0.263 μm per pixel. A manual quality control check was performed to rule out any cracks, bubbles, and physical damage to the slides before the slide digitization to ensure high quality digitized images. The digitized WSIs were output in SVS file format.

### Deep learning-based nuclei segmentation and cell type identification

The nuclei segmentation and cell type identification task was performed using TIAToolbox, a suite of tissue image analytic pipelines and libraries.^24^ TIAToolbox uses the HoVer-Net CNN architecture trained on multiple publicly available datasets, such as PanNuke and MoNuSAC.^10^, ^12^, ^29^ The PanNuke and MoNuSAC datasets include multiple images and tiles that have been curated and annotated with cell types.^10^, ^29^^,^ This facilitates training models on high quality data that can be used for predicting nuclei for a new WSI input. The HoVer-Net model pretrained on PanNuke dataset referred to as HoVer-Net(P) predicts epithelial cells (neoplastic and non-neoplastic), connective cells, inflammatory cells, and dead cells. The HoVer-Net model pretrained on MoNuSAC dataset (HoVer-Net(M)) predicts epithelial cells, lymphocytes, neutrophils, and macrophages.

All WSIs were processed for nuclei segmentation and cell type identification using the HoVer-Net(M) and HoVer-Net(P) models. The nuclei segmentation and cell-type predictions were performed on four Tesla V100 Graphical Processing Unit machines in parallel to accelerate the processing times.

### Integration of nuclei/cell segmentations from multiple model predictions

The predictions from the DL methods trained on the individual datasets were merged using the Annoy (Approximate Nearest Neighbors Oh https://github.com/spotify/annoy) algorithm. The implementation of this algorithm has been made available with in a GitHub repository with MIT licensing (https://github.com/jagadhesh89/MergeSegmentations). To ease installation for users, the algorithm has been made available as a python package, which installs all dependencies required. The aim of this novel integration approach is to yield a union of cell type predictions, while merging the overlapping nuclei and including the distinct non-overlapping nuclei predictions from the individual models. Annoy is a C++ library with Python bindings that enables rapid searching for points in space that are close to a given query point. Annoy uses random projections and trees to compute the nearest neighbor(s). Annoy splits the points in half recursively until each set has *n* items. Binary trees are built for every set of points and priority queues sorted by the minimum margin for the path from the root node are used to search the tree to get the nearest neighbors of a point. An Euclidean distance of 10 was chosen to define nearest neighbors based on profiling the distance of overlapping nuclei (Supplementary Fig. 1). The algorithm for merging of nuclei segmentations to derive cell type proportions were developed as follows:

**Inputs:**

• *M*_1_ = ${\left\{\left(x_{i}^{\left(1\right)},t_{i}^{\left(1\right)},p_{i}^{\left(1\right)},c_{i}^{\left(1\right)},b_{i}^{\left(1\right)}\;\right)\right\}}_{i=1}^{N_{1}}$: Set of nuclei from HoVer-Net(M)
  o $x_{i}^{\left(1\right)}\in R^{2}:\text{centroid position}$
  o $t_{i}^{\left(1\right)}\in T:\text{Predicted type from HoVer}-\mathrm{Net}\left(M\right)$
  o $p_{i}^{\left(1\right)}\in \left[01\right]:\text{Probability of prediction from HoVer}-\mathrm{Net}\left(M\right)$
  o $c_{i}^{\left(1\right)}\in R^{2}:\text{contour of nuclei HoVer}-\mathrm{Net}\left(M\right)$
  o $b_{i}^{\left(1\right)}\in R^{2}:\mathrm{box}\;\text{of nuclei HoVer}-\mathrm{Net}\left(M\right)$
• *M*_2_ = ${\left\{\left(x_{j}^{\left(2\right)},t_{j}^{\left(2\right)},p_{j}^{\left(2\right)},c_{j}^{\left(2\right)},b_{j}^{\left(2\right)}\;\right)\right\}}_{j=1}^{N_{2}}$: Set of nuclei from HoVer-Net(P)
  o $x_{j}^{\left(2\right)}\in R^{2}:\text{centroid position}$
  o $t_{j}^{\left(2\right)}\in T:\text{Predicted type from HoVer}-\mathrm{Net}\left(P\right)$
  o $p_{j}^{\left(2\right)}\in \left[01\right]:\text{probability of prediction from HoVer}-\mathrm{Net}\left(P\right)$
  o $c_{j}^{\left(2\right)}\in R^{2}:\text{contour of nuclei HoVer}-\mathrm{Net}\left(P\right)$
  o $b_{j}^{\left(2\right)}\in R^{2}:\mathrm{box}\;\text{of nuclei HoVer}-\mathrm{Net}\left(P\right)$
• *D* = 10: Euclidean distance threshold for merging nuclei

**Outputs:**

• *M* = $\left\{\left(x_{k},t_{k},p_{k},c_{k},b_{k}\right)\right\}:\text{Merged}\;\mathrm{set}\;\text{of nuclei predictions}$
• Counts and proportions of each type *t* per sample
• A comma separated value (.csv) file that shows report of nuclei that were merged and unmerged, including statistics of merge by cell type. (Example of this is shown in Supplementary Table 1)
• A comma separated value (.csv) file that shows report of equivocal nuclei, where the nuclei are overlapping, but are of different types across the predictions and are of high probability (>0.75) (Example of this is shown in Supplementary Table 2)

**Algorithm implementation:**

• **Initialize**
  o Set M ←∅
  o for each $n_{i}^{\left(1\right)}\in M_{1}$, set match($n_{i}^{\left(1\right)}$) ←false
  o for each $n_{i}^{\left(2\right)}\in M_{1}$, set match($n_{j}^{\left(2\right)}$) ←false
• **Build Annoy Index**
  o Build Annoy index *A* on the positions ${\left\{\left(x_{i}^{\left(1\right)}\;\right)\right\}}_{i=1}^{N_{1}}$.(n(trees) = 100, search_k = −1)
• **Merging algorithm**
for each nucleus $n_{j}^{\left(2\right)}=\left(x_{j}^{\left(2\right)},t_{j}^{\left(2\right)},p_{j}^{\left(2\right)},c_{j}^{\left(2\right)},b_{j}^{\left(2\right)}\;\right)\in M_{2}$
  o Find nearest neighbor $n_{i\left\{j\right)}^{\left(1\right)}=\left(x_{i\left(j\right)}^{\left(1\right)},t_{i\left(j\right)}^{\left(1\right)},p_{i\left(j\right)}^{\left(1\right)},c_{i\left(j\right)}^{\left(1\right)},b_{i\left(j\right)}^{\left(1\right)}\;\right)\;\mathrm{in}\;M_{1}\;\mathrm{using}\;A$
  o Compute distance $d=\|\;x_{j}^{\left(2\right)}-x_{i\left(j\right)}^{\left(1\right)}$||_2_
  o if d≤D
    ▪ if $t_{j}^{\left(2\right)}=t_{i}^{\left(1\right)}:$ **#Merge nuclei**
      • $x_{k}=\frac{x_{j}^{\left(2\right)}+x_{j}^{\left(1\right)}}{2}$
      • $t_{k}=t_{j}^{\left(2\right)}$
      • $p_{k}=\max\left(p_{j}^{\left(2\right)},p_{i}^{\left(1\right)}\;\right)$
      • $c_{k}=c_{j}^{\left(2\right)}$
      • $\;b_{k}=b_{j}^{\left(2\right)}$
      • $\mathrm{Add}\;\left(x_{k},t_{k},p_{k},c_{k},b_{k}\right)$ to *M*
      • $\mathrm{Set}\;\mathrm{match}\left(n_{j}^{\left(2\right)}\right)\leftarrow \mathrm{True}$
      • Set match($n_{i\left\{j\right)}^{\left(1\right)}$) ←True
    ▪ else $t_{j}^{\left(2\right)}\neq t_{i}^{\left(1\right)}$: **#Retain nucleus with higher probability**
      • if $p_{j}^{\left(2\right)}\geq p_{i}^{\left(1\right)}:$
        o $\mathrm{Add}\;n_{j}^{\left(2\right)}\;\text{to}\;M$
        o $\mathrm{Set}\;\mathrm{match}\left(n_{j}^{\left(2\right)}\right)\leftarrow \mathrm{True}$
      • else:
        o Add $n_{i\left\{j\right)}^{\left(1\right)}$ to *M*
        o Set match($n_{i\left\{j\right)}^{\left(1\right)}$) ←True
  o else *d* > *D*: **#Retain unmatched HoVer-Net(P)**
    ▪ $\mathrm{Add}\;n_{j}^{\left(2\right)}\;\text{to}\;M$
    ▪ $\mathrm{Set}\;\mathrm{match}\left(n_{j}^{\left(2\right)}\right)\leftarrow \mathrm{True}$
  for each $n_{i}^{\left(1\right)}\in M_{1}\text{where match}\left(n_{i}^{\left(1\right)}\right)=\text{False}$ **#Retain unmatched HoVer-Net(M)**
    ▪ Add $n_{i}^{\left(1\right)}$ to *M*
• **Compute cell type proportions**
for each type t∈T
  o count(*t*) = |{$n_{k}\in M\;|\;t_{k}=t$}|
  o proportion(*t*) = $\frac{\text{count}\left(t\right)\;}{\sum_{t^{\prime }\in T}\mathrm{count}\left(t^{\prime }\right)}$

### Comparison of cell type proportion estimates between the manual pathology review and deep learning models

The epithelial estimates available from the manual pathology review in deciles were compared with the proportion of epithelial cells from the individual DL-methods and the Annoy-based combined method using concordance correlation coefficient. The manual pathology review also included confirmation that lymphocytic population represented ≤2% of all cells.^27^ scipy (version 1.11.3 was used throughout the study) was used to perform two-sample Student's *t*-tests to compare means of epithelial estimates from the individual DL-methods, the Annoy-based integrated results, and the estimates from manual review.^30^ Further analysis was performed to verify if the lymphocytic proportions from the corresponding DL-based models and the integrated results were consistently ≤2% of all cells as determined by the manual review. Finally, a two-sample *t*-test was performed to compare mean lymphocytic proportion estimates between cases stratified by the presence/absence of higher proportion of TILs.

### Proportion of variance explained in bulk RNA expression by manual estimates and deep learning methods

To assess the potential superiority of DL-based cell-type proportion estimates relative to manual review, we assessed the respective associations with the corresponding RNA-Seq expression profiles. The primary objective was to determine which set of estimates better explained variation in the gene expression data, which would be reflective of better capturing the true underlying cell type distributions. Specifically, multiple gene-specific linear mixed models were built using variancePartition (1.30.2) package in R (4.3.1) to quantify variation in gene expression attributable to the estimates from the manual pathologist review and the individual models and the integrated results.^13^ The proportion of variance of bulk-RNA expression data explained by the manual estimates of epithelial and lymphocyte cells were compared with the estimates from DL-based cell proportion estimates. DL predictions from HoVer-Net(M) and HoVer-Net(P) and the integrated predictions using Annoy were modeled independently to evaluate the benefits of the merge over the independent predictions.

### Gene set enrichment analysis

Further examination of the relationship between DL-based proportions and expression was conducted using a subset of the genes (403 genes) with high explained variation (≥30%) by DL-based epithelial proportion. The analysis was performed using the WebGestaltR package (0.4.6) in R (4.3.1), with false-discovery rate method set as Benjamini–Hochberg with a value of <0.05.^34^ The enriched pathways/biological functions were carefully examined manually for relevance to the cell type.

## Results

### Integrated DL-based nuclei segmentations and cell type proportions

**Fig. 2:**
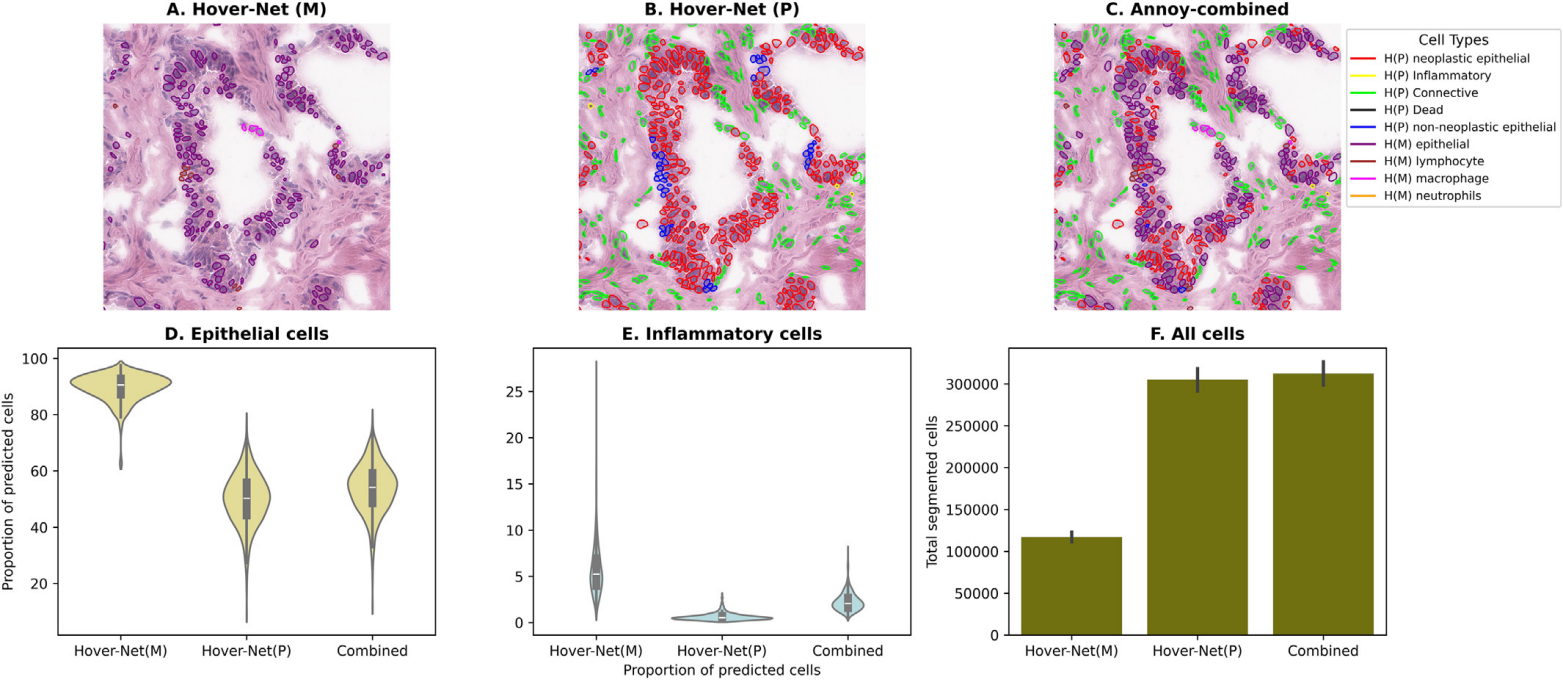
DL-based cell segmentation and Annoy-based combined nuclei/cell segmentation. 2A–C depicts a 1024 × 1024 tile from one of the 471 normal prostate samples to highlight segmentations as an example. It can be observed in 2C that the combined nuclei segmentation provides a comprehensive annotation compared to individual models in 2A and 2B. 2D depicts epithelial cell proportions from the models, and it demonstrates a higher distribution for HoVer-Net(M) model. 2E shows the distribution of inflammatory cells from the models, where it is observed that HoVer-Net(P) shows lowest distributions for this cell type. 2F demonstrates the comprehensiveness of accounting for distinct nuclei types that the Annoy-based combined results yield.

The algorithm for merging nuclei and calculating cell type proportions operates efficiently, with a runtime of under ten  minutes for each WSI. Fig. 2A–C demonstrates an example tile (a 1024 × 1024 pixel-sized image from the WSI) from one of the samples in our cohort. The Annoy-based combined results in Fig. 2C demonstrate the advantage of an integrated cell segmentation, where the approach integrates several types of nuclei predicted from the HoVer-Net(M) in Fig. 2A and the HoVer-Net(P) in Fig. 2B. While observing the predictions at the cell-type level, the median epithelial cell proportions across the samples for Annoy-based integrated methods, HoVer-Net(M), and HoVer- Net(P) were 50.27%, 90.54%, and 54.20%, respectively (Fig. 2D). The higher proportion of epithelial cells in HoVer-Net(M) was attributed to its lack of ability to segment connective cells, which on average constitutes 43% of cells on a WSI examined in our study. This results in elevated proportions of all other cell types in HoVer-Net(M). The median inflammatory cell proportions for the Annoy-based integrated methods, HoVer-Net(M), and HoVer-Net(P) were 2.08%, 5.24%, and 0.52%, respectively (Fig. 2E). The higher proportion of inflammatory cells from HoVer-Net(M) is again attributed to its lack of ability to segment connective cells, thereby, resulting in elevated proportions of all other cell types, and it was also noted that HoVer-Net(P) had a very low proportion of inflammatory cells owing to the training dataset not being comprehensively inclusive of different nuclei types for inflammatory cells. Finally, the combined method accounted for an average of 7305 additional cells being segmented over HoVer-Net(P) and 195,173 additional cells compared to HoVer-Net(M) (Fig. 2F).

### Comparison of DL cell proportion estimates to manual estimates

**Fig. 3:**
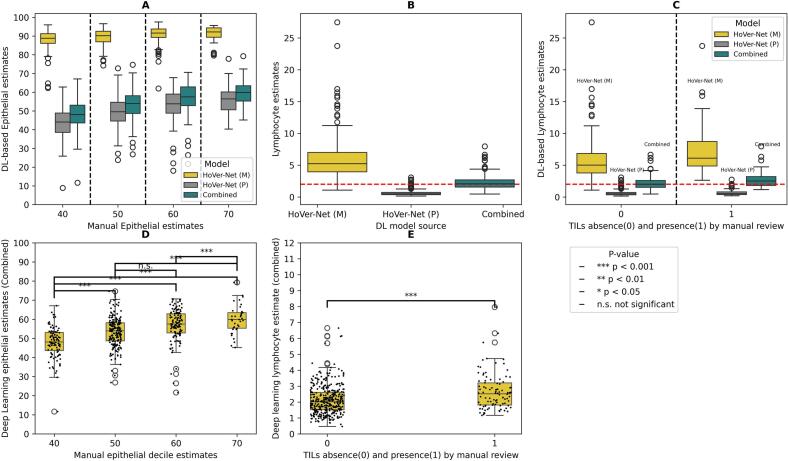
Evaluation of DL-based cell proportion estimates for epithelial cells and lymphocytes. 3A demonstrates the different distributions of epithelial proportions determined by the DL method when compared with deciles of epithelial cell proportion by manual review. 3B depicts lymphocyte distributions as predicted by HoVer-Net(M), HoVer-Net(P), and the Annoy-based combined prediction. The horizontal dashed red line in 3B–C indicates 2%, which was the threshold used in inclusion criteria of samples. 3C depicts the lymphocyte distribution broken down by absence/presence of TILs as indicated by manual pathologist review. 3D depicts the statistical significance for the distribution of combined annoy-based epithelial cell proportions compared against manual epithelial estimates. 3E depicts the statistical significance for the distribution of combined DL-based lymphocyte cell proportions as per-presence and absence of higher proportion of TILs. (For interpretation of the references to color in this figure legend, the reader is referred to the web version of this article.)

The Annoy-based combined method's epithelial cell proportion estimates demonstrated the highest concordance when compared to epithelial estimates from manual pathologist review. Among the individual DL-methods, we observed that the HoVer-Net(M) showed the lowest concordance to the manual epithelial estimates. The concordance correlation coefficients of comparing the epithelial cell proportion estimates to manual pathologist review were 0.012 [0.006,0.017] for HoVer-Net(M), 0.407 [0.330,0.478] for HoVer-Net(P) and 0.412 [0.333,0.479] for Annoy-based combined method. Fig. 3A presents distributions of epithelial cell proportion by the DL-method and deciles of epithelial cell proportion reported by manual pathologist review. The proportion of epithelial cells derived from the Annoy-based integrated predictions and HoVer-Net(P) trended similarly to the estimates from manual pathologist review, whereas the HoVer-Net(M) was observed to predict a higher proportion of epithelial cells due to lack of ability to segment connective cells, hence over-estimating other cell type proportions.

Based on the careful pathology review as described in methods, we expect the lymphocytic cell proportions to be ≤2% of all cells in our sample cohort. Table 1 demonstrates the median estimates of lymphocytic cells from the individual models and the integrated method. We observed that lymphocytic cell proportions derived from Annoy-based combined predictions showed a median value of 2.0817 [range = 2.0039,2.1635], consistent with the manual pathologist review (Table 1). Fig. 3B indicates the lymphocytic distributions from the corresponding methods. When the lymphocytic distribution was stratified by the presence/absence of TILs, we observed that the cases where the pathologist indicated presence of higher proportion of TILs had higher DL-derived lymphocyte distributions (Fig. 3C).

**Table 1:** Median estimates of lymphocytic cell distributions of the DL-based methods.

Method	Median
HoVer-Net(M)	5.2400 [5.0200–5.4700]
HoVer-Net(P)	0.5173 [0.4981–0.5479]
Combined (Annoy-based)	2.0817 [2.0039–2.1635]

The epithelial cell proportions derived from Annoy-based combined predictions demonstrated distinct distributions stratified by the deciles of manual epithelial estimates (Fig. 3D). Pairwise testing *p*-values were <0.05, except for the 50 vs. 60 comparison (*P* = 0.06). The lymphocyte cell proportions derived from combined Annoy-based predictions also demonstrated a statistically different distribution (*P* < 0.001) when stratified by the presence/absence of higher TILs (Fig. 3E).

### Proportion of variance of bulk-RNA expression explained by DL and manual methods

**Fig. 4:**
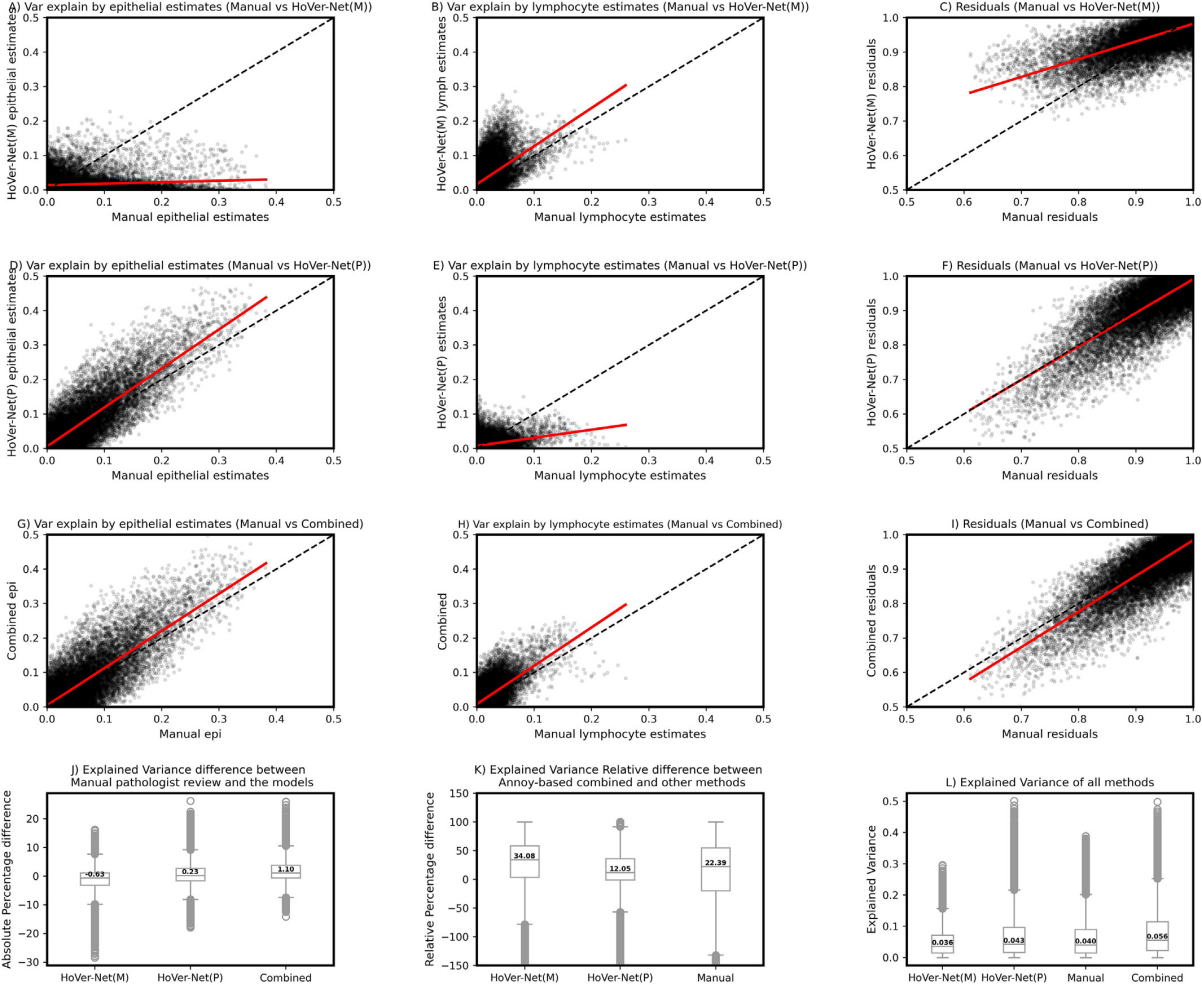
Linear mixed models to characterize the bulk RNA expression profiles with estimates of cell proportion from DL and manual methods yielded proportion of explained variance. The explained variance is compared in this analysis between the manual review and the DL methods. HoVer-Net(M), HoVer-Net(P) and the combined annoy-based predictions are compared with the manual review to understand the proportion of explained and the unexplained variance. HoVer-Net(M) explained the variance in bulk RNA expression well for lymphocyte estimates compared to manual review but failed to explain the variance based on epithelial estimates (4A–B). HoVer-Net(P) explained the variance in bulk RNA expression well for the epithelial cell estimates but performed lower compared to manual review for the lymphocytes (4D–E). In both models, HoVer-Net(M) and HoVer-Net(P), unexplained variance was lower when compared with the manual method (4C and 4F). However, the combined nuclei segmentation results when merged between DL methods trained on both datasets, the proportion of explained variance of the expression profiles was higher for both the cell types and unexplained variance for the DL method was lower than the manual method (4G, 4I). Quantitively, the Annoy-based combined method showed highest explained variance when compared to manual method (explained variance is calculated as (1-residual variance), and difference of explained variance between each model and manual method is computed on per-gene basis) (4J). A positive improvement in explained variance was observed when compared to the other methods indicating the superiority of the Annoy-based method (4K). The median explained variance of the Annoy-based combined method was the highest compared to other methods (4L).

The cell proportion estimates from HoVer-Net(M) and HoVer- Net(P) showed lower explained variance of the RNA expression on average for specific nuclei type(s) when used in a stand-alone mode. For instance, the epithelial estimates from the HoVer-Net(M) model explained a lower proportion of expression variance compared to the manual epithelial estimates in 67.5% of the genes (Fig. 4A), however, it showed the ability to explain higher variance of the expression compared to manual estimates for lymphocytes in 67.8% of the genes (Fig. 4B). Also, the residuals (unexplained variance) based on the HoVer-Net(M) model tended to be higher compared to the manual estimates for the cell types in 59.5% of the genes (Fig. 4C). Likewise, the epithelial cell proportion estimates from the HoVer-Net(P) model explained a higher proportion of expression variance compared to the manual epithelial estimates in 57.6% of the genes (Fig. 4D), however, it lacked the ability to explain the variance based on lymphocyte estimates compared to the manual review in 57.34% of the genes (Fig. 4E). The residual (i.e., unexplained) variance based on the HoVer-Net(P) model tended towards the diagonal with 46.5% of genes showing higher residual variance compared to the manual estimates (Fig. 4F). Finally, when the nuclei segmentation results were merged based on Annoy algorithm, it was observed that the proportion of variance explained by both the epithelial and lymphocyte estimates from this method were higher than the manual pathologist's estimates in 54.1% and 62.5% of the genes, respectively (Fig. 4G–H) and also the unexplained variance by the DL method for the cell type estimates were lower compared to the manual review, with the residual variance from the combined method higher than manual in only 34.1% of the genes (Fig. 4I). The Annoy-based combined method showed the highest explained variance compared to manual pathologist estimates; the median of percentage difference in explained variance between manual and Annoy-based combined method was 1.10, 0.23 for HoVer- Net(P) and −0.063 for Hover-Net(M) (Fig. 4J). The variance explained on a per gene basis was compared between the Annoy-based combined method and other methods; these showed a median of 34% improvement over HoVer- Net(M), 12.05% improvement over HoverNet(P), and 22.39% improvement over manual estimates (Fig. 4K). Finally, the median variance of the expression explained by Annoy-based combined segmentation for the 24,075 genes was 0.056 (Inter Quartile Range (IQR): [0.023,0.115]), which is 26.2% more than HoVer-Net(P) (median: 0.043, IQR:[0.016,0.096]), 43.48% more than HoVer-Net (M) (median:0.036, IQR:[0.015,0.072]), and 33.33% (median: 0.040, IQR: [0.015,0.090]) more than the manual estimates, thereby, indicating an overall enhancement of ability to explain expression while accounting for various cell types in the Annoy-based integrated method (Fig. 4L).

### Gene set enrichment analysis

**Fig. 5:**
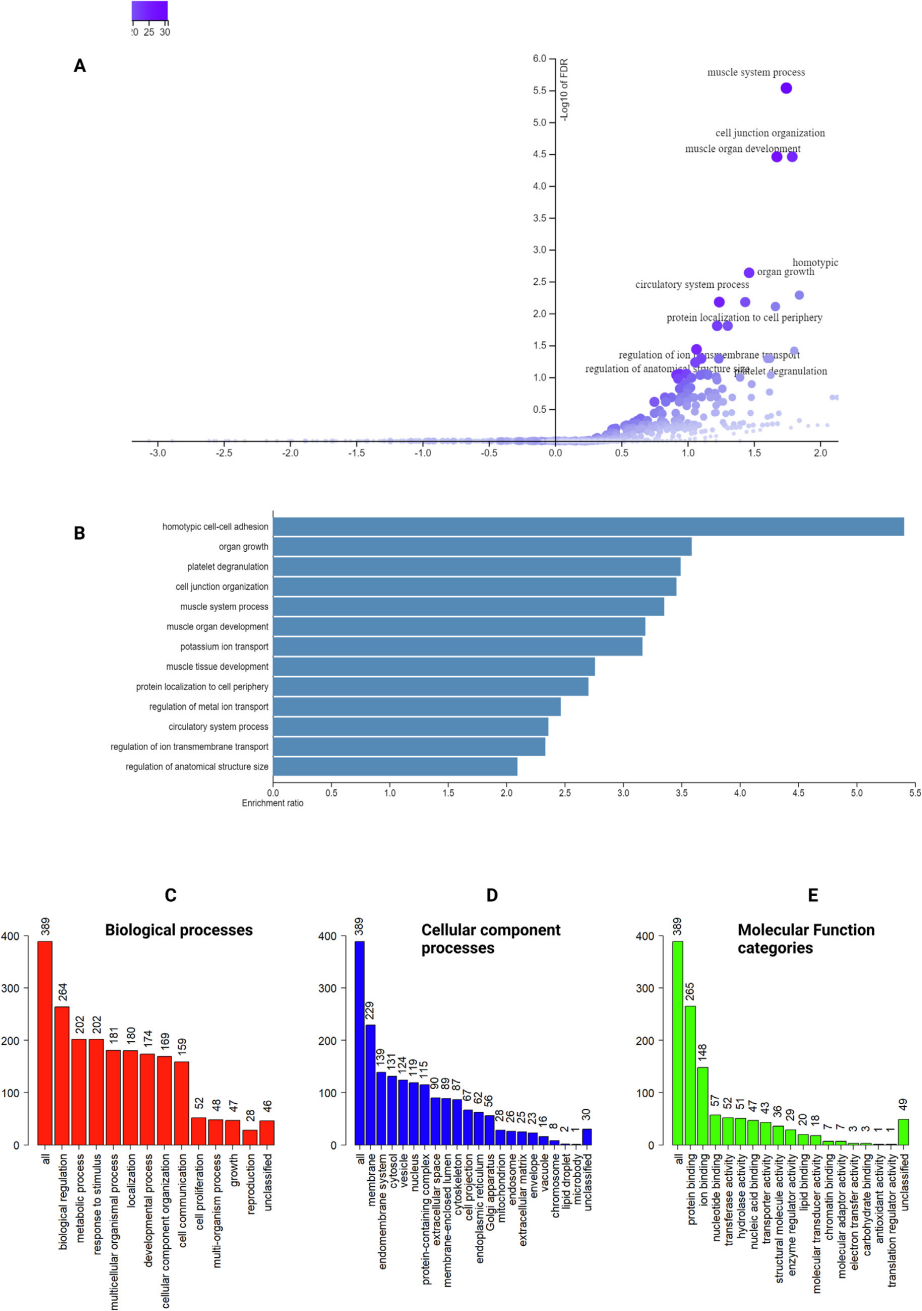
GSEA indicated relevant biological process for genes explained by high variance proportion (5A–C). 5A indicates the relevant biological pathways as plotted by the enrichment ratio and the FDR, 5B indicates a bar plot of the biological process by enrichment ratio and 4C indicates the proportion of the 403 genes that explain specific relevant biological pathways. 5D indicates a closer look at the cellular component process that indicates relevant membrane and endomembrane functions and 5E indicates relevant ion and protein binding and transport processes.

Subsequently, the gene-set enrichment analyses on 403 genes with high explained variation (≥30%) by Annoy-based combined estimated epithelial proportions was performed and the results demonstrated enrichment of highly relevant biological pathways. The top few biological processes identified by the gene set enrichment analyses were homotypic cell–cell adhesion, organ growth, platelet degranulation, smooth muscle process, muscle organ development, and cell junction organization (Fig. 5A–B). It has been previously established that smooth muscle participates in reciprocal signaling with epithelium to generate complex patterns of epithelial folds.^11^ In addition, the epithelial cells participate in creating cell–cell junctions that help maintain homeostasis by regulating the structural integrity of the tissues, the diffusion of ions, solutes, and microbes across the tissue, cell proliferation, and cell migration.^14^ The cell–cell junctions that the epithelial cells create line the organs and help in organ development, organ growth, maintaining the homeostasis.^31^ Studies have also established the role between epithelial cells and platelet degranulation indicating that direct signaling between platelets and cancer cells induces an epithelial–mesenchymal-like transition that promotes metastasis.^19^ Closer examination of the cellular component process highlighted by enrichment analysis confirmed that a higher proportion of the genes enriched towards membrane and the endomembrane processes (Fig. 5C–D), and epithelial cells' role in endomembrane transport processes and polarity has been previously demonstrated.^14^, ^22^, ^31^^,^ Finally, the study of these genes enriched in terms of molecular function categories indicated the protein and ion binding processes as the top results (Fig. 5E) and these processes have been established as key functions of epithelial cells 14, 19, 22, 31, 32.

## Discussion

H&E-stained pathology slides offer means to study cell types and their proportions.^12^ Pathologists often perform a H&E slide review to obtain crude estimates of cell types and proportions.^12^ However, manual reviews are often tedious, time-consuming, and are prone to human errors, affecting reproducibility.^12^ Digitization of the H&E slides to obtain WSIs, an inexpensive solution, offers a rich data modality for exploration using multiple computational approaches. However, DL-based methods and its derived solutions are often viewed as black-box approaches and multiple efforts are underway to improve the reliability and forge trust in the underlying methods.^1^ Also, DL-based methods are often limited by their ability to generalize and wide-applicability owing to limitations in the training datasets.^9^

In our study, we explore popular DL-based methods trained on robust labeled datasets to identify cell types on WSIs for deriving cell proportions by type. This inexpensive alternative approach to derive cell type proportions is fast, scalable, and can yield key information towards various downstream applications such as understanding tumor microenvironment. In addition to demonstrating the feasibility of the DL-based methods for the nuclei segmentation, our study underpins the reliability of the derived estimates from DL-based methods. By examining the nuclei segmentation results from DL-based methods trained on individual datasets independently and carefully comparing the DL-derived cell proportions with a diligent manual pathologist review, our study outlines the limitations of the individual models (owing to the distributions in training sets) and highlights the need for an integrated and a comprehensive approach. The comprehensive integrated approach should account for diverse set of nuclei to accurately compute the cell type proportions. By comparing the cell type proportions trained on individual models and our comprehensive approach, we underscore the need for accounting for nearly every nuclei on a WSI and show that lack of such approach might result in incorrect representation of cell type proportions. Our study further demonstrates the feasibility of using the DL-derived epithelial and lymphocyte proportions towards a bulk-RNA expression deconvolution approach by assessing the explained variance in the bulk-RNA expression attributable to the DL-based cell type proportions. The results from the variance analyses further highlights the limitations of relying heavily on cell proportion estimates from a single model trained on a single dataset, where we observed that the DL-based cell proportion estimates from HoVer-Net(P) and HoVer-Net(M) failed to explain the variance in the bulk-RNA expression data for specific cell types. This observation further bolstered the need for methods to integrate the predictions from the DL-based approaches. Previous studies that explored merging the nuclei predictions from DL-based approaches trained on multiple datasets included computationally expensive training approaches that are resource heavy^38^. We have addressed this limitation by proposing an efficient and a novel approach capable of integrating nuclei segmentation from multiple DL-based methods and computing cell type proportions within ten  minutes per WSI. We have demonstrated the comprehensive ability of this integrated approach to account for distinct cell types. The ensemble approach proposed in our study is based on a nearest-neighbors approach that does not require model training, thereby reducing the need for resource heavy data management and computing. In addition, the cell type proportions derived from the integrated cell segmentation explained the variance of bulk-RNA expression better than the manual and individual-model approaches for both epithelial and lymphocyte cell types, demonstrating the robustness of the approach. Finally, the gene-set enrichment analysis using the genes that explained high variance (≥30%) emphasized that the genes were enriched for relevant biological functions and pathways, thereby establishing reliability of the integrated cell type proportions. In summary, our approach is easy to install, implement and provides a time-efficient means to merge the cell segmentations with no additional model training involved.

There has been increased interest in the digital pathology field to efficiently use the bulk-RNA data in conjunction with H&E slides for various applications.^4^, ^7^, ^8^, ^15^, ^16^, ^25^, ^37^ Notably, there has been recent high-impact research that uses cascaded diffusion models to generate synthetic H&E tumor slides using bulk-RNA expression data using HoVer-Net(P) for training/prediction for cell segmentations.^5^ We believe that such methods and outcomes would be further enhanced when combining cell/nuclei predictions from multiple models as outlined in our study.

Our future work will involve using the reliable integrated cell proportions towards a bulk-RNA expression deconvolution approach to derive gene expression by cell types. Although there are multiple DL methods that have been outlined to infer the gene expression from WSIs, it is notable that these studies infer the bulk RNA gene expression and lack ability to outline the expression by cell types.^2^, ^25^, ^37^ A recent study outlined a method (SCHAF) to infer cell proportions and gene expression by the cell types using H&E slides, however, this method works on the assumption that there is availability of both imaging data and scRNA-seq data for the model training.^6^ It is also notable that SCHAF might have limitations with regards to being generalizable and applicability of the method to other datasets need further study.^6^ In efforts to study the cell type proportions and the expression by cell types, current expression deconvolution approaches involve using the bulk-RNA and the scRNA sequencing techniques to develop probabilistic computational approaches,^4^, ^7^, ^8^, ^15^, ^16^ however, these methods are limited by the need of high-quality samples, analytical challenges, and prohibitive costs. By establishing the feasibility and reliability of DL-based integrated cell type proportions, our study provides an inexpensive alternative that leverages DL-based cell proportions and bulk-RNA expression to derive gene expression by cell type.

A potential limitation of our study is the variety of cell types that can be evaluated using the DL-based methods. Currently, the methods evaluated in our study can segment epithelial cells (neoplastic and non-neoplastic), lymphocytes, neutrophils, macrophages, inflammatory cells, connective cells, and dead cells. The ability to segment other specific cell types or sub-types of cell types is limited by current available annotated datasets. Availability of such wider annotated datasets and advanced DL methods trained on them can foster improvement. The merging algorithm outlined requires careful consideration of nuclei types that are available, hence our current method offers ability to merge nuclei and assign cell types based on popular public datasets that includes PanNuke and MoNuSAC. Although merging of nuclei and assigning cell types from more than two models is computationally possible, there is a requirement for a schema accounting for alignment of cell type assignments across the methods and defining the final output category. We have carefully assigned the cell types and output categories in our algorithm (Supplementary Table 3) and we emphasize the need for such carefully designed user-mapping for expanding this method for merging across more than two methods. Alternatively, this schema could be represented as a tree structure in a manner that child nodes in the tree are always consistent with parent nodes upstream in the tree structure, which would automatically handle any number of different types of classifications so long as the cell-types are represented in the tree. Another challenge in our study was related to the manual pathology review of the slides for estimating the epithelial cell proportion and presence of TILs. Owing to the scale of thousands of nuclei present in a single slide, the pathologist provided crude estimates in deciles, and this might have resulted in approximation of the cell proportions. For instance, it is notable that the epithelial proportion from DL estimates were not statistically significant between 50% and 60% as reported by the manual pathology (*P*-value = 0.06, Fig. 2), and this was a result of approximation in the manual pathology review. Despite this challenge, it was notable that the distributions of estimates from the DL methods showed statistically significant differences when compared to all the other levels as demonstrated by the results. Finally, there exists a fraction of challenging nuclei that are overlapping across the predictions where the cell types of nuclei do not match. In a small portion of these overlapping unmatched cell types of nuclei, the probabilities can be high across both the models (>0.75) owing to the training sets of the corresponding models. We handle this challenge by assigning the cell type based on highest probability, and also generate a sample-level report on these subset of nuclei assigned as equivocal that includes the centroid position and the types called by the corresponding models (Supplementary Table 2). We anticipate that this feature will facilitate a decision-support process involving a manual review for the challenging nuclei and also offer improvement opportunities for future methods.

In conclusion, our study emphasizes the utility in integrating cell segmentations from multiple DL-methods, offers a novel solution for the integration, thoroughly validates the approach, and establishes feasibility of using the derived cell proportions towards a potential bulk-RNA expression deconvolution approach. Finally, we anticipate that our results will stimulate the development of alternative approaches to leverage and integrate multiomics/multimodal data involving H&E slide digitization and bulk RNA gene expression.

## Funding

This work was funded by grants from the 10.13039/100000005DOD (W81XWH-11-1-0261) and from the US Public Health Service and National Institutes of Health (CA151254, CA157881 and CA015083).

## Declaration of competing interest

The authors declare the following financial interests/personal relationships which may be considered as potential competing interests:

Nicholas B. Larson reports financial support was provided by US Department of Defense. Nicholas B. Larson reports financial support was provided by National Institutes of Health. If there are other authors, they declare that they have no known competing financial interests or personal relationships that could have appeared to influence the work reported in this article.

## Data Availability

The WSIs used in this study will be available upon request. The code and the data used to perform the analyses is available on GitHub under MIT license: (https://github.com/jagadhesh89/MergeSegmentations).

## References

[1] Ali S. (2023). Explainable artificial intelligence (XAI): what we know and what is left to attain trustworthy artificial intelligence. Inform Fusion.

[2] Alsaafin A. (2023). Learning to predict RNA sequence expressions from whole slide images with applications for search and classification. Commun Biol..

[3] Anders S., Pyl P.T., Huber W. (2015). HTSeq—a Python framework to work with high-throughput sequencing data. Bioinformatics.

[4] Avila Cobos F. (2020). Benchmarking of cell type deconvolution pipelines for transcriptomics data. Nat Commun.

[5] Carrillo-Perez F., Pizurica M., Zheng Y. (2025). Generation of synthetic whole-slide image tiles of tumours from RNA-sequencing data via cascaded diffusion models. Nat. Biomed. Eng.

[6] Charles C. (2023). Inference of single cell profiles from histology stains with the Single-Cell omics from Histology Analysis Framework (SCHAF). bioRxiv.

[7] Chu T. (2022). Cell type and gene expression deconvolution with BayesPrism enables Bayesian integrative analysis across bulk and single-cell RNA sequencing in oncology. Nat Cancer.

[8] Cobos F.A. (2023). Effective methods for bulk RNA-seq deconvolution using scnRNA-seq transcriptomes. Genome Biol.

[9] Freiesleben T., Grote T. (2023). Beyond generalization: a theory of robustness in machine learning. Synthese.

[10] Gamper, J., et al. PanNuke: an open pan-cancer histology dataset for nuclei instance segmentation and classification, Digital Pathology. 11–19.

[11] Garcia, M.A., Nelson, W.J. and Chavez, N. Cell-Cell Junctions Organize Structural and Signaling Networks. LID - 10.1101/cshperspect.a029181 LID - a029181. (1943–0264 (Electronic)). PMC5773398 28600395

[12] Graham S. (2019). Hover-Net: simultaneous segmentation and classification of nuclei in multi-tissue histology images. Med Image Anal.

[13] Hoffman G.E., Schadt E.E. (2016). variancePartition: interpreting drivers of variation in complex gene expression studies. BMC Bioinform.

[14] Ikenouchi, J.A.-O. Roles of Membrane Lipids in the Organization of Epithelial Cells: Old and New Problems. (2168–8370 (Electronic)). 10.1080/21688370.2018.1502531 PMC6179127 30156967

[15] Im, Y.A.-O. and Kim, Y.A.-O. A Comprehensive Overview of RNA Deconvolution Methods and Their Application. (0219–1032 (Electronic)). 10.14348/molcells.2023.2178 PMC9982058 36859474

[16] Jew B. (2020). Accurate estimation of cell composition in bulk expression through robust integration of single-cell information. Nat Commun.

[17] Kalari K.R. (2014). MAP-RSeq: Mayo analysis pipeline for RNA sequencing. BMC Bioinform..

[18] Kong, Y., et al. Nuclear Segmentation in Histopathological Images Using Two-Stage Stacked U-Nets With Attention Mechanism. (2296–4185 (Print)). 10.3389/fbioe.2020.573866 PMC7649338 33195135

[19] Labelle, M., Begum S.F., Hynes, R.O. Direct Signaling Between Platelets and Cancer Cells Induces an Epithelial-Mesenchymal-Like Transition and Promotes Metastasis. (1878–3686 (Electronic)). 10.1016/j.ccr.2011.09.009 PMC3487108 22094253

[20] Langmead B. (2009). Ultrafast and memory-efficient alignment of short DNA sequences to the human genome. Genome Biol.

[21] Mahmood F.F., Borders, D., et al. Deep Adversarial Training for Multi-Organ Nuclei Segmentation in Histopathology Images. (1558-254X (Electronic)). 10.1109/TMI.2019.2927182 PMC8588951 31283474

[22] Mysior, M.A.-O. and Simpson, J.A.-O. Cell3: A New Vision for Study of the Endomembrane System in Mammalian Cells. LID - 10.1042/BSR20210850C LID - BSR20210850C. (1573–4935 (Electronic)).

[23] Newman, A.A.-O., et al. Determining Cell Type Abundance and Expression from Bulk Tissues with Digital Cytometry. (1546–1696 (Electronic)). 10.1038/s41587-019-0114-2 PMC6610714 31061481

[24] Pocock J. (2022). TIAToolbox as an end-to-end library for advanced tissue image analytics. Commun Med.

[25] Schmauch B. (2020). A deep learning model to predict RNA-Seq expression of tumours from whole slide images. Nat Commun.

[26] Schmidt U. (2018). Lecture Notes in Computer Science.

[27] Thibodeau S.N. (2015). Identification of candidate genes for prostate cancer-risk SNPs utilizing a normal prostate tissue eQTL data set. Nat Commun.

[28] Trapnell C. (2012). Differential gene and transcript expression analysis of RNA-seq experiments with TopHat and Cufflinks. Nat Protoc.

[29] Verma R. (2021). MoNuSAC2020: a multi-organ nuclei segmentation and classification challenge. IEEE Trans Med Imaging.

[30] Virtanen P. (2020). SciPy 1.0: fundamental algorithms for scientific computing in Python. Nat Methods.

[31] Volckaert, T. and De Langhe, S. Lung Epithelial Stem Cells and Their Niches: Fgf10 Takes Center Stage. (1755–1536 (Print)). 10.1186/1755-1536-7-8 PMC4041638 24891877

[32] Vrana, N.E., et al. Engineering Functional Epithelium for Regenerative Medicine and In Vitro Organ Models: A Review. (1937–3376 (Electronic)). 10.1089/ten.teb.2012.0603 PMC3826472 23705900

[33] Wagner, A., Regev, A. and Yosef, N. Revealing the Vectors of Cellular Identity with Single-Cell Genomics. (1546–1696 (Electronic)). 10.1038/nbt.3711 PMC5465644 27824854

[34] Wang J. (2017). WebGestalt 2017: a more comprehensive, powerful, flexible and interactive gene set enrichment analysis toolkit. Nucleic Acids Res.

[35] Wang L., Wang S., Li W. (2012). RSeQC: quality control of RNA-seq experiments. Bioinformatics.

[36] Weigert M. (2020).

[37] Xiao X. (2024). Transformer with convolution and graph-node co-embedding: an accurate and interpretable vision backbone for predicting gene expressions from local histopathological image. Med Image Anal.

[38] Zhang W. (2023). Merging nucleus datasets by correlation-based cross-training. Med Image Anal.

