# Supplementary material for: Efficient merging and validation of deep learning-based nuclei segmentations in H&E slides from multiple models
Source: J Pathol Inform. 2025 Apr 15;17:100443. doi: 10.1016/j.jpi.2025.100443 (PMC12130990; doi:10.1016/j.jpi.2025.100443)
Supplement: Supplementary material [file mmc1.docx]

## Appendix A: Supplementary Information


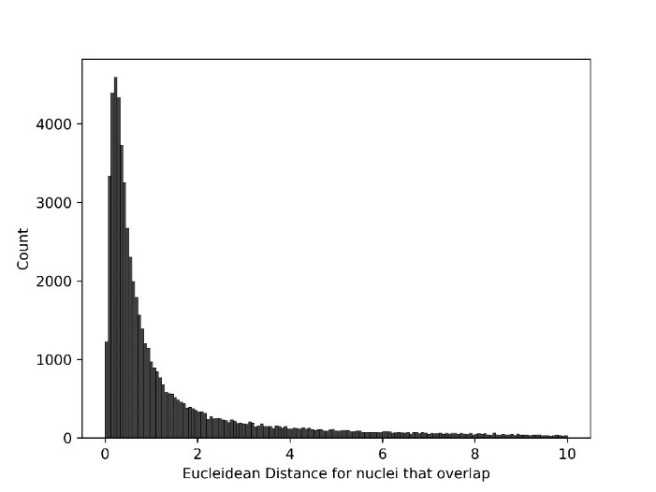


Figure S1. Euclidean distances were profiled for overlapping nuclei. It can be observed that all of the overlapping nuclei were within 10 distance units, with majority of them being 2 or less.

Supplementary Table 1: Merging report sample

| **Contributions** | **Total** | **Description** |
| --- | --- | --- |
| Mon_merges | 9028 | Overlapping nuclei where types did not match between the two predictions, and MoNuSac cell type was chosen based on higher probability |
| Pan_merges | 12367 | Overlapping nuclei where types did not match between the two predictions, and PanNuke cell type was chosen based on higher probability |
| Mon_Pan_merges | 45429 | Cell types from the two models matched on overlapping nuclei and they were successfully merged |
| Unmerged_from_pan | 99611 | Non-overlapping unique PanNuke based nuclei |
| Unmerged_from_mon | 5344 | Non-overlapping unique MoNuSac based nuclei |
| Merged_nuclei | 66824 | Number of nuclei that overlapped and merged between the datasets |
| Total_Ensemble_Nuclei | 171779 | Total nuclei in the final combined result. |
| Equivocal Nuclei percentage | 5.1% | Percentage of nuclei that overlap, cell types don’t match and probabilities of assignment is >0.75 |

Supplementary Table 2: Sample of Equivocal merges – Overlapping nuclei where cell types do not match and both models show high probability (>0.75).

| PanNuke centroid (x-y) | MoNuSac centroid (x-y) | PanNuke cell type | MoNuSac cell type | PanNuke probability | MoNuSac probability |
| --- | --- | --- | --- | --- | --- |
| 19114.15-10048.39 | 19115.12-10050.69 | connective | lymphocyte | 0.975 | 0.893 |
| 19143.61-10064.50 | 19141.92-10066.16 | connective | lymphocyte | 0.970 | 0.830 |

Supplementary Table 3: User-assigned mapping for ensemble cell type assignment for nuclei that overlap

| HoVer-Net (M) | HoVer-Net (P) | Ensemble method output |
| --- | --- | --- |
| Epithelial | Neoplastic epithelial | Epithelial |
| Lymphocyte | Inflammatory cell | Lymphocyte |
| Macrophage | Inflammatory cell | Macrophage |
| Neutrophil | Inflammatory cell | Neutrophil |
| Epithelial | Non-neoplastic epithelial | Epithelial |
